# Exploring ceRNA networks for key biomarkers in breast cancer subtypes and immune regulation

**DOI:** 10.1038/s41598-023-47816-z

**Published:** 2023-11-27

**Authors:** Alireza Shariatmadar Taleghani, Yasaman Zohrab Beigi, Fatemeh Zare-Mirakabad, Ali Masoudi-Nejad

**Affiliations:** 1 Laboratory of Systems Biology and Bioinformatics (LBB), Institute of Biochemistry and Biophysics, University of Tehran, Tehran, Iran; 2 Department of Mathematics and Computer Science, Amirkabir University of Technology (Polytechnic Tehran), Tehran, Iran

**Keywords:** Computational biology and bioinformatics, Biomarkers, Breast cancer

## Abstract

Breast cancer is a major global health concern, and recent researches have highlighted the critical roles of non-coding RNAs in both cancer and the immune system. The competing endogenous RNA hypothesis suggests that various types of RNA, including coding and non-coding RNAs, compete for microRNA targets, acting as molecular sponges. This study introduces the Pre_CLM_BCS pipeline to investigate the potential of long non-coding RNAs and circular RNAs as biomarkers in breast cancer subtypes. The pipeline identifies specific modules within each subtype that contain at least one long non-coding RNA or circular RNA exhibiting significantly distinct expression patterns when compared to other subtypes. The results reveal potential biomarker genes for each subtype, such as circ_001845, circ_001124, circ_003925, circ_000736, and circ_003996 for the basal-like subtype, circ_00306 and circ_00128 for the luminal B subtype, circ_000709 and NPHS1 for the normal-like subtype, CAMKV and circ_001855 for the luminal A subtype, and circ_00128 and circ_00173 for the HER2+ subtype. Additionally, certain long non-coding RNAs and circular RNAs, including RGS5-AS1, C6orf223, HHLA3-AS1, circ_000349, circ_003996, circ_003925, circ_002665, circ_001855, and DLEU1, are identified as potential regulators of T cell mechanisms, underscoring their importance in understanding breast cancer progression in various subtypes. This pipeline provides valuable insights into cancer and immune-related processes in breast cancer subtypes.

## Introduction

Breast cancer continues to hold its position as the prevailing type of cancer, constituting a significant portion of diagnoses among women globally, with approximately one in three cases being attributed to this malignancy. Statistical data from 2021 reveal that an estimated 281,550 new cases were diagnosed; accounting for 14.8% of all cancer diagnoses, and tragically, breast cancer was responsible for 43,600 deaths, representing 7.2% of the total cancer-related fatalities^1^. Characterized by its immunohistochemical properties, hormone receptors, and specific protein involvement, breast cancer is classified into five primary molecular subtypes: basal-like, luminal B, normal-like, luminal A, and HER2+^2^. These subtypes exhibit significant differences in characteristics^3^. Although substantial advancements have been made in clinical treatment, their efficacy could be further improved with a more precise understanding of the distinguishing features and molecular mechanisms associated with each breast cancer subtype^4,5^. This knowledge could significantly influence treatment outcomes and patient survival rates^6^.

Non-coding RNAs (ncRNAs) play pivotal roles in the regulating of gene expression^7^. The ncRNAs include long ncRNAs (lncRNAs), microRNAs (miRNAs), and circular RNAs (circRNAs). MiRNAs are short, single-stranded ncRNA molecules. They primarily modulate gene expression by binding to target mRNAs leading to their degradation or repression of translation^8^. LncRNAs are a broad class of ncRNAs exceeding 200 nucleotides in length. They regulate mRNA production by influencing transcription of protein-coding genes^9^. Moreover, lncRNAs may affect tumorigenesis by controlling key cancer-related genes^10^. CircRNAs are a relatively new class of endogenous small ncRNAs. Unlike most RNAs, they lack both a 5′ cap and 3′ poly-A tail, typically resulting from splicing errors^11^. Advances in RNA sequencing technologies and bioinformatics have accelerated research on circRNAs, highlighting their significant role in gene expression regulation, primarily by acting as miRNA sponges^12^. Similar to miRNAs and lncRNAs, circRNAs have been linked to various complex human diseases^13,14^, including several types of cancers^15^. Their dysregulation and potential functional roles make circRNAs promising candidates for further exploration and potential therapeutic interventions in cancer research. While the roles of miRNAs, lncRNAs, and circRNAs in cancer pathology and physiology are increasingly being elucidated, their interaction within competing endogenous RNAs (ceRNA) networks promises new insights into their regulatory mechanisms^16^. Some studies suggest that ceRNAs are pivotal to many vital biological processes and could serve as potential diagnostic markers, prognostic indicators, or therapeutic targets^17,18^. The regulatory influence of miRNAs goes beyond their capacity to recognize target sites on various RNA molecules^19^. It has been suggested that miRNAs can facilitate regulatory crosstalk among different components of the transcriptome^20^. This intricate miRNA-mediated regulation is modulated by other RNA molecules, including mRNAs and ncRNAs, which have been found to interact with miRNAs and exhibit significant expression across diverse biological conditions. This mechanism introduces an additional layer of post-transcriptional gene regulation, providing a complementary perspective on the functional relevance of the vast number of transcribed, yet un-translated RNAs. The concept of ceRNAs further enhances this post-transcriptional regulatory landscape, where ncRNAs gain new significance. The interplay mediated by miRNAs among different types of RNA molecules has been observed in numerous contexts^21,22^.

The ceRNAs hypothesis posits that a single miRNA can regulate multiple target RNAs, as long as they contain the specific miRNA response element. This hypothesis introduces the notion that RNAs can compete for a limited pool of miRNAs, serving as the foundation for constructing a ceRNA network^23^. Recently, the emergence of public databases has facilitated ceRNA network reconstruction, shedding light on the previously elusive mechanisms of tumorigenesis in cancers, such as breast cancer^24^.

Although previous studies have separately analyzed lncRNA–miRNA–mRNA^25^ and circRNA–miRNA–mRNA^26^ ceRNA networks, no research to date has integrated both circRNAs and lncRNAs into the ceRNA network for the basal-like, luminal B, normal-like, luminal A, and HER2+ breast cancer subtypes. Examining ceRNA networks across these subtypes, and combining circRNAs and lncRNAs, can significantly enhance our understanding of immune-related tumorigenesis in various breast cancer subtypes.

In this study, we present a novel pipeline, called Pre_CLM_BCS, designed to identify potential circRNAs, lncRNAs, and mRNAs as effective biomarkers for each breast cancer subtype. Our pipeline initiates by constructing a primary ceRNA network for each cancer subtype using gene expression data from patients. Subsequently, specific ceRNA networks are extracted for each breast cancer subtype from their respective primary ceRNA networks. Within these specific networks, modules containing at least one lncRNA or circRNA are identified and enriched with gene ontology terms and pathways. Finally, we employ survival analysis and differential gene expression to predict biomarkers from the enriched genes in the modules, which are further evaluated by support vector machine.

The predicted biomarkers for each breast cancer subtype according to the pipelines are as follows:

- For the basal-like subtype: circ_001845, circ_001124, circ_003925, circ_000736, and circ_003996.
- For the luminal B subtype: circ_00306 and circ_00128.
- For the normal-like subtype: circ_000709 and nephrotic syndrome 1 (NPHS1 as an mRNA).
- For the luminal A subtype: cam kinase like vesicle associated (CAMKV as an mRNA) and circ_001855.
- For the HER2+ subtype: circ_00128 and circ_00173.

Additionally, we find the following RNAs in modules that have regulatory effects on T cell mechanisms, further emphasizing the significance of these RNAs in understanding and addressing breast cancer progression across various subtypes:

- For the basal-like subtype: circ_003996, and circ_003925.
- For the luminal B subtype: chromosome 6 open reading frame 223 (C6orf223 as an lncRNA), HHLA3 Antisense RNA 1 (HHLA3-AS1 as an lncRNA), and circ_000349.
- For the normal-like subtype: deleted in lymphocytic leukemia 1 (DLEU1 as an lncRNA).
- For the luminal A subtype: circ_002665 and circ_001855.
- For the HER2+ subtype: regulator of G protein signaling 5 antisense RNA 1 (RGS5-AS1 as an lncRNA).

## Materials and methods

**Figure 1:**
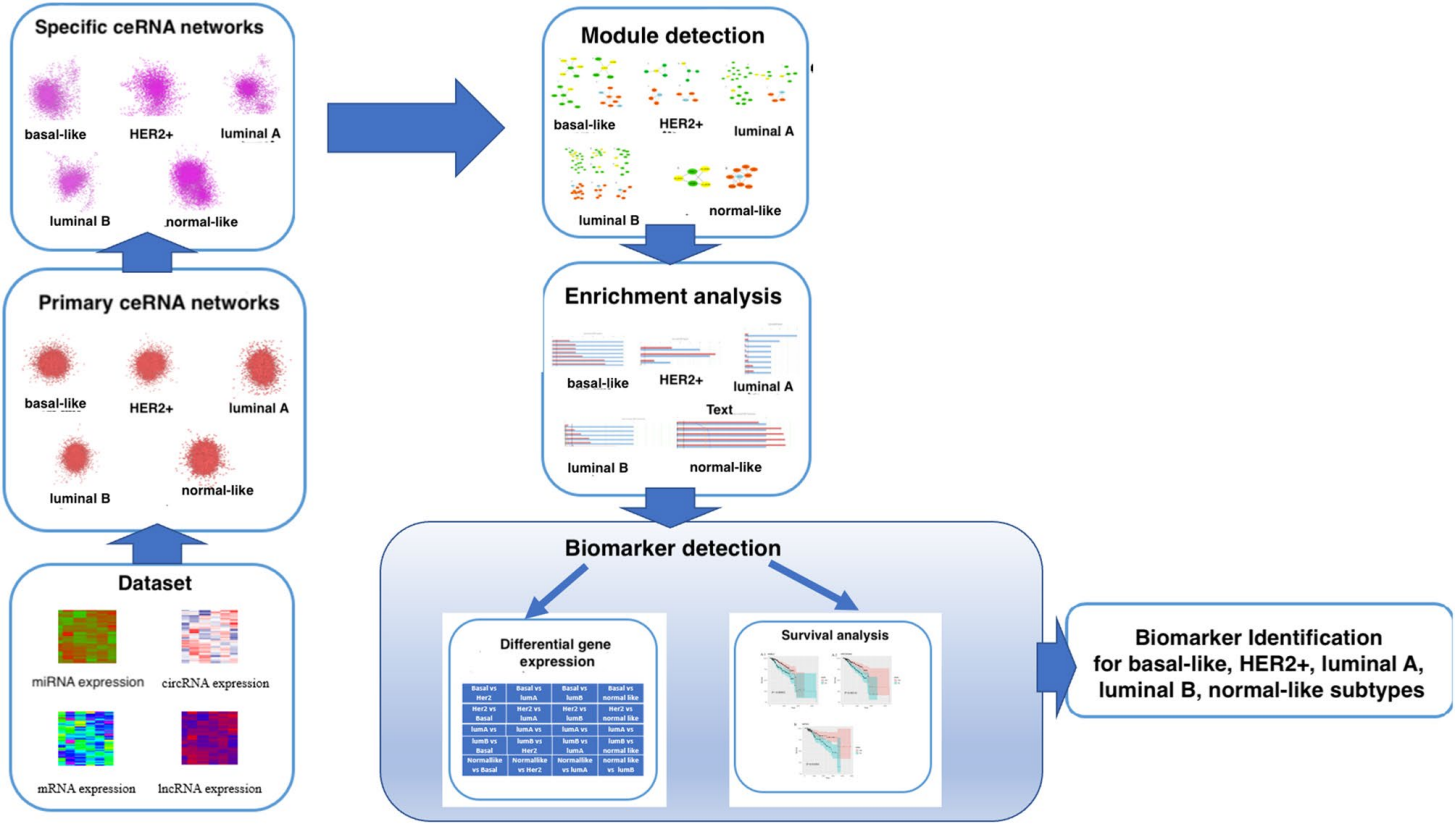
The Pre_CLM_BCS pipeline.

In this section, our main goal is to introduce the proposed pipeline, Pre_CLM_BCS, for predicting suitable circRNAs, lncRNAs, and mRNAs as biomarkers for five breast cancer subtypes: basal-like, luminal B, normal-like, luminal A, and HER2+. Figure 1 visually outlines our pipeline, and the following subsections offer in-depth explanations for each step of the process.

### Dataset

Breast cancer is traditionally classified into five subtypes as follows:

$$BCS = \left\{ {basal{-}like, luminal\;B, normal{-}like, luminal\;A, HER2 + } \right\}.$$

In this study, we extract miRNA, mRNA, and lncRNA gene expression data, along with clinical data for breast cancer samples from The Cancer Genome Atlas (TCGA) database (as of August 2015).

We utilize the miRNA-seq dataset for miRNA gene expression and the RNA-SeqV2 level 3 dataset for mRNA and lncRNA expression profiles. To annotate the mRNA and lncRNA genes from the RNA-SeqV2 level 3 dataset, we use the BioMart tool available on the Ensembl website (www.ensembl.org) as a genome browser^27^. Concurrently, circRNA expression data for breast cancer subtypes is sourced based on the findings of Asha et al.^28^.

Additionally, we collect clinical data, including immunohistochemical information, for breast cancer samples from TCGA database, in order to conduct survival analysis.

Considering the conventional categorization of breast cancer into five subtypes, our study samples are categorized accordingly. This results in the creation of five distinct datasets for mRNA, lncRNA, circRNA, and miRNA. For each cancer subtype, we establish a dataset denoted as $\Delta^{C}$, where $C \in BCS$.

Each dataset encapsulates the gene expression of mRNAs, lncRNAs, circRNAs, and miRNAs for samples within the respective breast cancer subtypes. The data preprocessing for each dataset $\Delta^{C}$ comprises three main stages:

1. Elimination of genes with a mean expression value below 0.5.
2. Exclusion of genes with uniform expression across all five breast cancer subtypes, indicating low variance.
3. Detection of outliers. To identify outliers, our pipeline utilizes hierarchical clustering of the samples based on their expression profiles within the R environment (version 3.6)^29^.

### Primary ceRNA networks

In the second step of the Pre_CLM_BCS pipeline, we construct a primary ceRNA network named $$P^{C} = \left( {N^{C} ,E^{C} } \right)$$ for each breast cancer subtype $$C \in BCS$$ using gene expression data from dataset $$\Delta^{C}$$. Here, $$N^{C} $$ represents the collection of mRNAs, lncRNAs, and circRNAs in the dataset as nodes. The set of edges (interactions) in this network is defined as $$ E^{C}$$, where each edge between two RNAs from set $$N^{C}$$ indicates their interaction^30^. To identify these interactions, we utilize the Pearson correlation coefficient (PCC) for the gene expression dataset $$\Delta^{C}.$$

Our approach for constructing a primary ceRNA network is rooted in the ceRNA regulatory model. According to this model, lncRNAs and circRNAs compete with miRNAs, resulting in opposing effects on mRNA expression^31,32^. Therefore, when the expression of an miRNA increases, there is a corresponding decrease in the levels of lncRNAs, mRNAs, and circRNAs. This competition weakens the positive correlation among miRNAs, lncRNAs, and mRNAs^33,34^.

Consequently, a simultaneous increase in mRNA expression along with either lncRNA or circRNA implies the potential downregulation of a specific miRNA. To explore interactions as edges in the primary ceRNA network $P^{C}$ among lncRNAs and mRNAs, we utilize the miRTarBase and TarBase databases to access experimentally verified mRNA–miRNA interactions.^35,36^ We also extract experimentally verified mRNA–miRNA interactions from the miRTarBase database.

To predict interactions as edges among the union of mRNAs and lncRNAs in the set $N^{C}$, following the ceRNA regulatory model, we compute the PCC on the gene expression data of the RNAs, extracting their interactions from the miRTarBase and TarBase databases. In other words, assuming there is a documented interaction between miRNA A and both mRNA B and lncRNA C in the databases, we predict an edge between mRNA B and lncRNA C if the gene expression data shows a negative PCC between miRNA A and mRNA B, as well as between miRNA A and lncRNA C. Simultaneously, there should be a positive PCC between the gene expression data of mRNA B and lncRNA C. Therefore, in cases where lncRNAs and miRNAs share a common miRNA, as indicated in the mentioned databases, we forecast potential interaction between them.

Due to the absence of experimental interaction data between circRNAs and miRNAs, we predict interactions among circRNAs or between circRNAs and mRNAs solely based on the PCC. In other words, to infer the interactions between circRNAs and mRNAs, we calculate the PCC for each pairing of circRNAs with miRNAs, mRNAs with miRNAs, and circRNAs with mRNAs. We predict an interaction between a circRNA and an mRNA when there is a positive PCC between their gene expression data, as well as a negative PCC between the gene expression data of the circRNA and a miRNA, as well as between the mRNA and that miRNA.

For both the scenarios: negative PCC values between microRNAs with lncRNAs, mRNAs, and circRNAs, and positive PCC values among lncRNAs, mRNAs, and circRNAs pairings, a stringent threshold of an absolute PCC surpassing 0.4 and a *p *value beneath 0.05 is instated for statistical robustness. Meanwhile, we remove RNAs from the set $N^{C}$ if they lack edges connecting them to other RNAs.

In culmination, the visual representation of the primary ceRNA network $P^{C}$ for each $C \in CBCS$ can be achieved via Cytoscape^37^.

### Specific ceRNA networks

In the third step of pipeline, we aim to establish a specific ceRNA network for each breast cancer subtype to delve into the distinctive regulatory mechanisms inherent to each subtype. To achieve this, we first extract a common ceRNA network from the primary ceRNA networks. Subsequently, we derive the specific ceRNA network for each subtype, C, by subtracting the common ceRNA network from the primary ceRNA network $P^{C} = \left( {N^{C} ,E^{C} } \right)$.

To achieve this, we start by constructing the common ceRNA network called $cCENT = \left( {N^{cCENT} ,E^{cCENT} } \right)$ by amalgamating the primary ceRNA networks, $P^{basal{-}like}$, $P^{luminal\;B}$, $P^{normal{-}like}$, $P^{luminal\;A}$ and $P^{HER2 + }$ where

$$ \begin{aligned} N^{cCENT} & = N^{basal{-}like} \cap N^{luminal\;B} \cap N^{normal{-}like} \cap N^{luminal\;A} \cap { }N^{HER2 + } , \\ E^{cCENT} & = E^{basal{-}like} \cap E^{luminal\;B} \cap E^{normal{-}like} \cap E^{luminal\;A} \cap { }E^{HER2 + } . \\ \end{aligned} $$

Then, a specific ceRNA network named $sCENT^{C} = \left( {N_{sCENT}^{C} ,E_{sCENT}^{C} } \right)$ is defined for each breast cancer subtype $C \in BCS$ as bellow:

$$ \begin{aligned} N_{sCENT}^{C} & = N^{C} - N^{cCENT} , \\ E_{sCENT}^{C} & = E^{C} - E^{cCENT} , \\ \end{aligned} $$

where $N^{C}$ and $E^{C}$ represent the number nodes (RNAs) and edges (interactions) among RNAs in the corresponding primary ceRNA network of breast cancer subtype C, respectively.

### Module detection

In the fourth step of the Pre_CLM_BCS pipeline, we employ ClusterMaker Cytoscape application^38^ to detect modules within the specific ceRNA networks. This application leverages the Markov cluster algorithm^39^. For each breast cancer subtype $C \in BCS$, the specific ceRNA network $sCENT^{C}$ is input into the application to identify modules. We select a collection of modules named $LC - MODUL^{C}$ that contains one lncRNA or circRNA for further analysis in breast cancer subtype $C$.

### Enrichment analysis

In the fifth step of the pipeline, potential Gene Ontology (GO) terms of the modules in the set $LC - MODUL^{C}$ of each breast cancer subtype, $C \in BCS$, are determined through GO enrichment analysis conducted using the ToppFun web tool. GO terms with FDR-corrected *p *values less than 0.05 are considered significant^40^.

### Biomarker detection

In the sixth step of our pipeline, we apply two methodologies to extract biomarkers from the set $$LC\_Module^{C}$$, focusing on breast cancer subtype $$ C$$. In the first methodology, we utilize survival analysis to identify effective mRNAs in breast cancer subtype $$C$$. In the second one, we employ differential gene expression data to predict biomarkers by comparing breast cancer subtype $$C$$ to the other subtypes separately.

#### Survival analysis

For each breast cancer subtype, denoted as $C \in BCS$, we calculate the union of mRNA genes from the modules listed in $LC\_Module^{C}$. Subsequently, we explore the relationship between the expression of these mRNA genes and patients' overall survival by employing the Kaplan–Meier (KM) estimation and Log-rank test. TCGA survival data matching the mRNA genes are retrieved from TCGA database for survival analysis. A total of 88, 100, 76, 252, and 42 samples are collected for basal-like, luminal B, normal-like, luminal A, and HER2+ subtypes, respectively. For each gene’s survival analysis, patients are divided into two groups based on their expression values. Those with values exceeding the mean are categorized as the high-expression group, while those with values below the mean are classified as the low-expression group. We compute Kaplan–Meier curves and Log-rank test statistical results for both groups using the Survminer^41^ and survival^42^ packages in R.

#### Differential gene expression analysis

For the analysis of differentially expressed genes (DEG), we create the set of genes named $G^{C}$ from $LC\_Module^{C}$ for each breast cancer subtype $C \in BCS$. Then, we conduct DEG analysis for each pair of subtypes $C,C^{\prime} \in BCS$, where $C \ne C^{\prime},$ as the between-subtype differentially expressed genes, denoted as $BSDEG^{C,C^{\prime}}$. This analysis is performed using the edgeR package on the gene expression data of the genes in $G^{C}$ ^43^. Each gene with adjusted *p *values less than 0.05 (Benjamini–Hochberg) and an absolute logFC value greater than 0.5 is included in the set $BSDEG^{C,C^{\prime}}$. Subsequently, we extract the set of biomarkers ($Bio\_DEG^{C}$) for breast subtype $C$, as follows:

$$ Bio\_DEG^{C} = \bigcap\limits_{\begin{subarray}{l} C^{\prime} \in BCS \\ C^{\prime} \ne C \end{subarray} } {BSDEG^{{C,C^{\prime}}} } . $$

To validate the predicted biomarkers in $Bio\_DEG^{C} { }$ for each cancer subtype $C$, we employ a support vector machine (SVM) classifier, and implement fivefold cross-validation. The validation procedures using the SVM classifier and fivefold cross-validation are performed individually for each subtype against all other subtypes. This process is repeated for each of the five subtypes.

After running SVM on the breast cancer subtype $C \in BCS$, we calculate the receiver operating characteristic (ROC) curve and the area under the curve (AUC) to evaluate the efficiency of the biomarkers, $Bio\_DEG^{C}$, in distinguishing the breast cancer subtype from the others. The caret^44^, plotROC^45^, MLmetrics^46^, and ggplot^47^ R packages are utilized to implement the SVM algorithm and to calculate and illustrate the ROC curves.

## Results

In this section, we offer a step-by-step representation of the results obtained from executing the pipeline outlined in Fig. 1 for biomarker detection to distinguish breast cancer subtypes.

### Dataset

**Figure 2:**
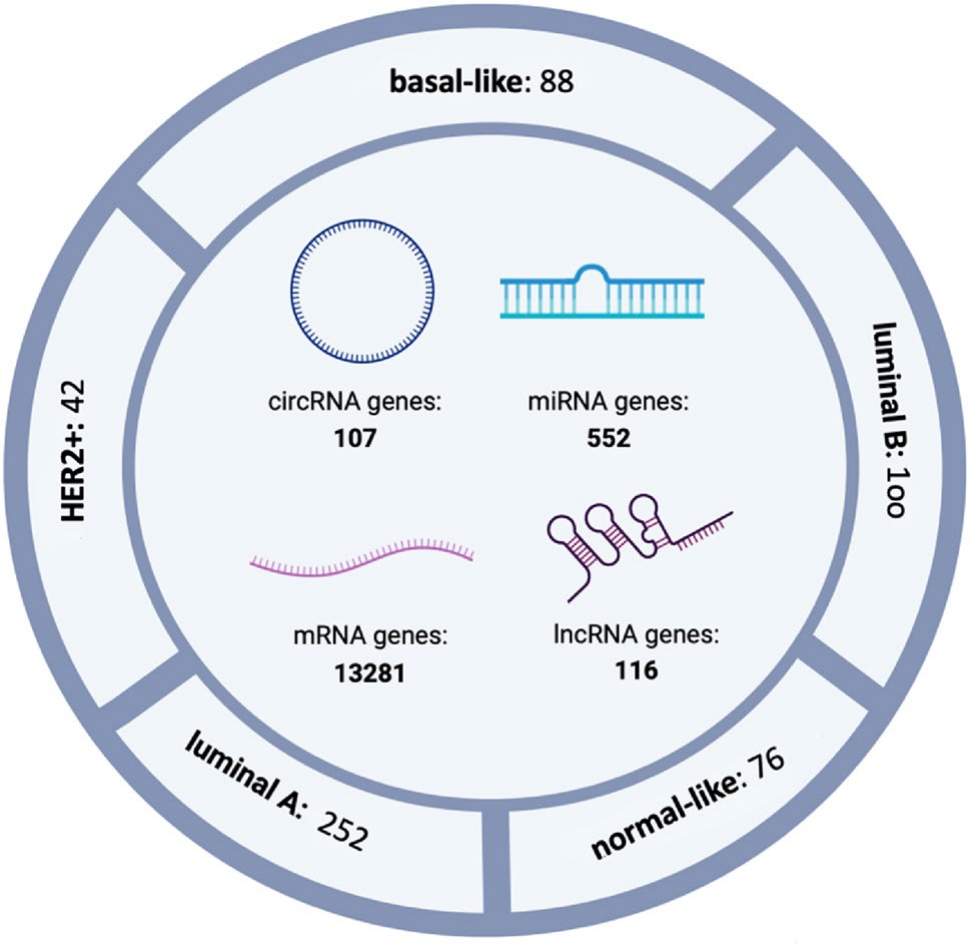
The number of genes and samples for breast cancer subtypes after preprocessing: basal-like, luminal B, normal-like, luminal B, and HER2+. *Note*: The picture was created using biorender software (biorender.com).

Figure 2 illustrates the outcomes of the initial step in the Pre_CLM_BCS pipeline. During this phase, we extract gene expression of 30,480 genes, encompassing mRNA, miRNA, circRNA, and lncRNA. After the completion of the first and second data preprocessing stages within this step, we narrow down the initial gene pool to 14,056 genes. The figure also breaks down the retained genes by their respective types.

Furthermore, after the third data preprocessing step, we remove105 out of 193 samples for the basal-like subtype, 119 out of 219 for luminal B, 67 out of 143 for the normal-like, 329 out of 581 for luminal A and 40 out of 82 for HER2+ subtypes of breast cancer as outlier data. Finally, for each subtype $C \in BCS$, we have a dataset as $\Delta^{C}$ which includes gene expression data of 14,056 genes and $n$ samples. The value of $n$ varies depending on the cancer type. Specifically, for basal-like, luminal B, normal-like, luminal A, and HER2+ subtypes, we found 88, 100, 76, 252, and 42 samples, respectively. Supplementary Figure S1 exhibits dendrograms illustrating the hierarchical clustering of samples conducted for outlier detection.

### Primary ceRNA networks

**Figure 3:**
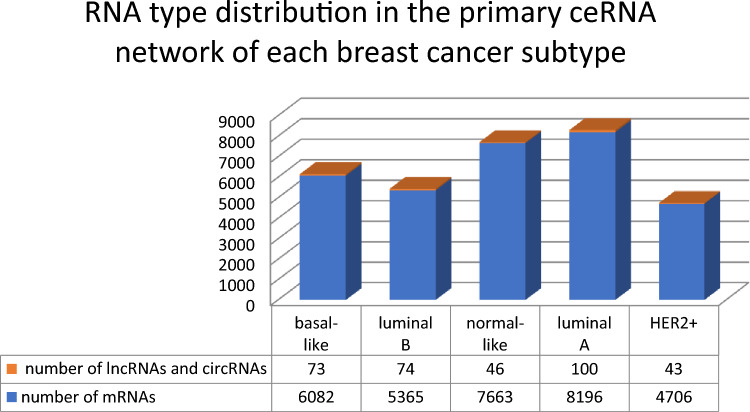
RNA type distribution across breast cancer subtypes.

We define $P^{C} = \left( {N^{C} ,E^{C} } \right)$ to represent each subtype $C \in BCS$ as the primary ceRNA network using the dataset $\Delta^{C}$. Table 1 shows the number of RNAs ($|N^{C} |$) and the number of edges among RNAs ($\left| {E^{C} } \right|$) for each subtype $C \in BCS$. Figure 3 illustrates the presence of mRNAs, lncRNAs and circRNAs in the primary ceRNA of each breast cancer subtype.

**Table 1:** The number of RNAs ($\left| {N^{C} } \right|$) and the number of edges among RNAs ($\left| {E^{C} } \right|$) for each subtype $C \in BCS$ in the primary ceRNA network $P^{C} = \left( {N^{C} ,E^{C} } \right)$.

$C$	Basal-like	Luminal B	Normal-like	Luminal A	HER2+
$|N^{C} |$	6155	5439	7709	8296	4749
$|E^{C} |$	368,773	261,344	724,686	676,146	169,644

### Specific ceRNA networks

**Figure 4:**
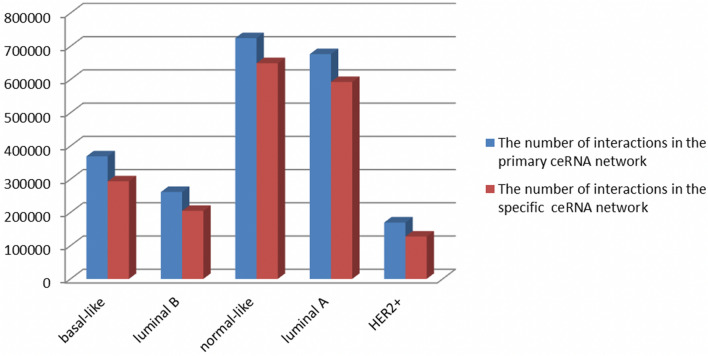
Comparison of the number of edges (interactions) in the specific and primary ceRNA networks for each breast cancer subtype.

For each breast cancer subtype $C \in BCS$, the specific ceRNA network named $sCENT^{C} = \left( {N_{sCENT}^{C} ,E_{sCENT}^{C} } \right)$ is derived by removing the common ceRNA network ($cCENT$) from the primary ceRNA network $P^{C}$. After removing the $cCENT$ network from the primary ceRNA network, we lose 8, 11, 2, 3 and 13 RNAs from the corresponding specific ceRNA networks for basal-like, luminal B, normal-like, luminal A and HER2+, respectively. Figure 4 compares the number of edges in $sCENT^{C}$ ($|E_{sCENT}^{C}$|) with the edges in the primary ceRNA network $P^{C}$ for each breast cancer subtype $C \in BCS$.

### Module detection

**Figure 5:**
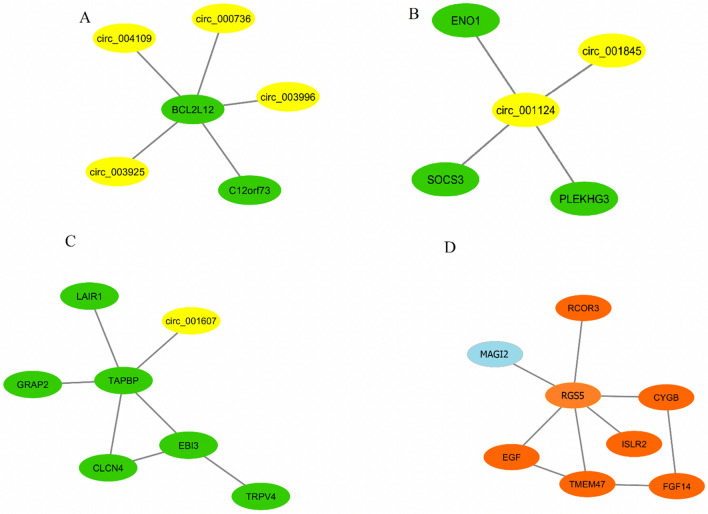
The basal-like subtype includes three distinct circRNA modules (**A**–**C**) and one lncRNA module (**D**). In A, B and C, the circRNAs and mRNAs are shown by yellow and green, respectively. In D, the lncRNA and mRNAs are shown by blue and brown, respectively.

**Figure 6:**
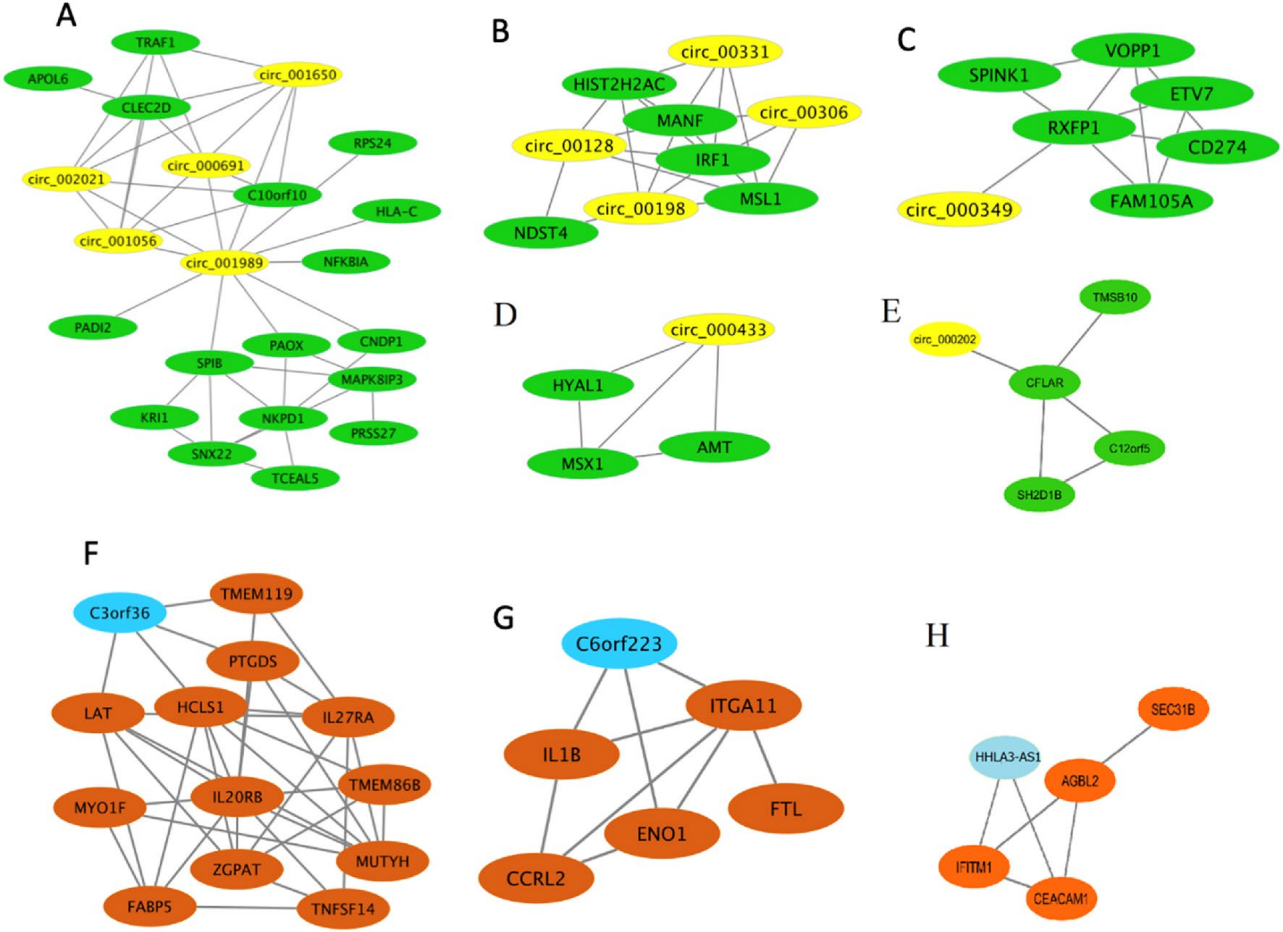
The luminal B subtype includes five distinct circRNA modules (**A**–**E**) and three lncRNA modules (**F**–**H**). In (**A**–**E**), the circRNAs and mRNAs are shown by yellow and green, respectively. In (**F**–**H**), the lncRNAs and mRNAs are shown by blue and brown, respectively.

**Figure 7:**
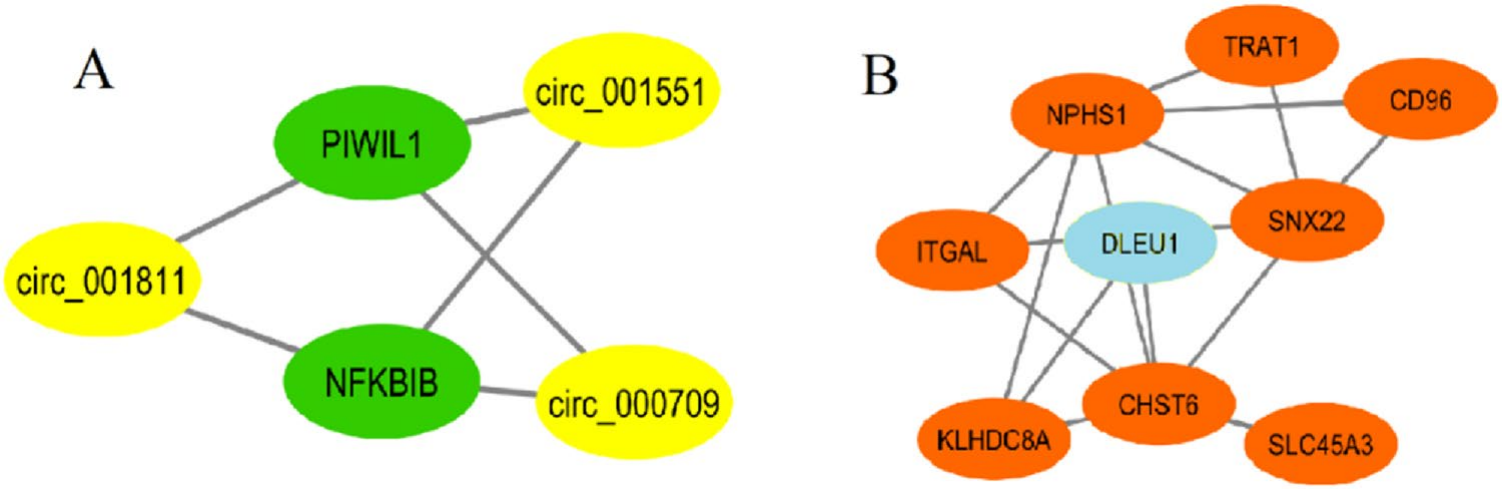
The normal-like subtype includes one circRNA module (**A**) and one lncRNA module (**B**). In (**A**), the circRNAs and mRNAs are shown by yellow and green, respectively. In (**B**), the lncRNAs and mRNAs are shown by blue and brown, respectively.

**Figure 8:**
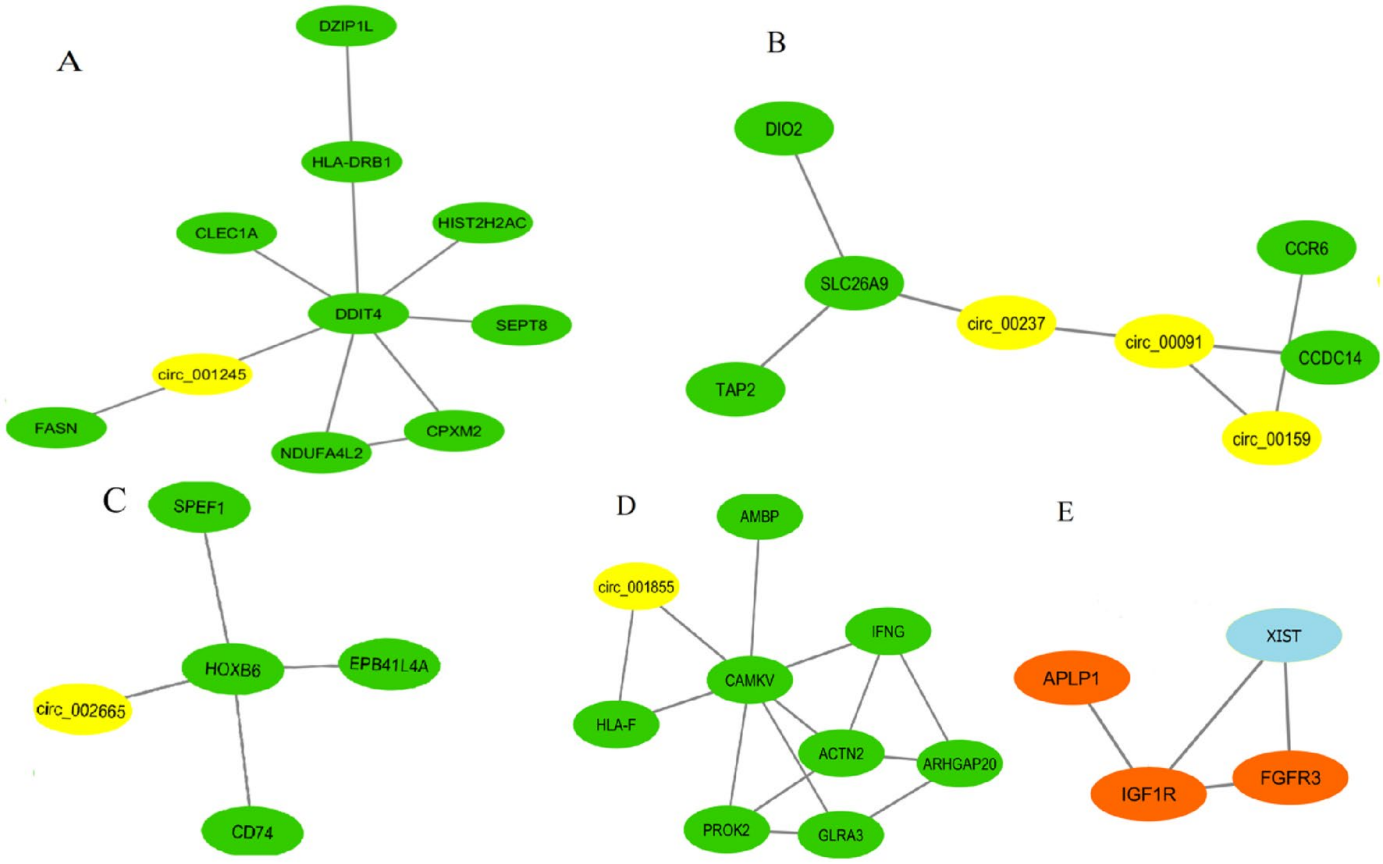
The luminal A subtype includes four distinct circRNA modules (**A**–**D**) and one lncRNA module (**E**). In (**A**–**D**), the circRNAs and mRNAs are shown by yellow and green, respectively. In (**E**), the lncRNA and mRNAs are shown by blue and brown, respectively.

**Figure 9:**
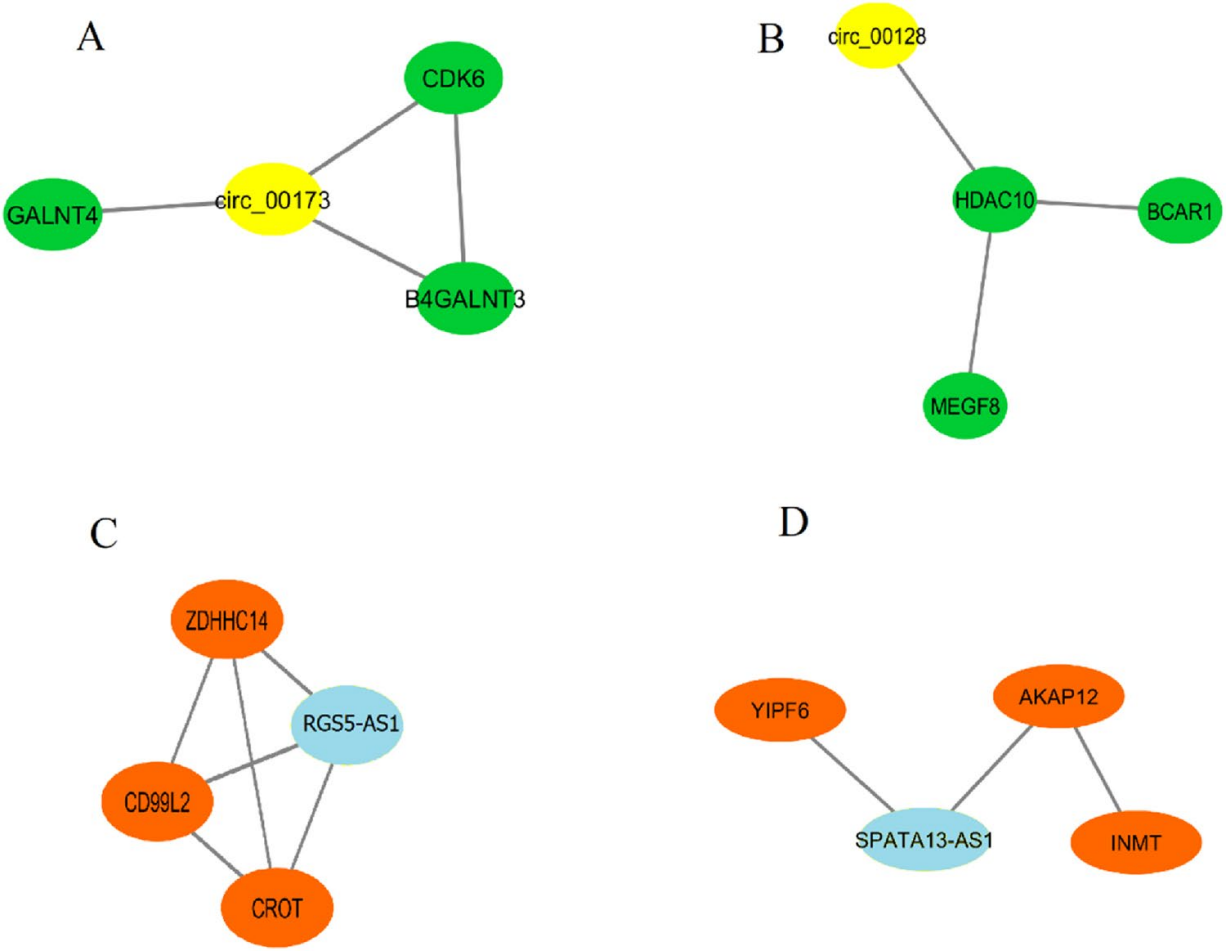
The HER2+ subtype includes two distinct circRNA modules (**A**,**B**) and two lncRNA modules (**C**,**D**). In (**A**,**B**), the circRNAs and mRNAs are shown by yellow and green, respectively. In (**C**,**D**), the lncRNAs and mRNAs are shown by blue and brown, respectively.

We select a set of modules as $LC - MODUL^{C}$ including at least one lncRNA or circRNA. Figures 5, 6, 7, 8, and 9 depict these modules for basal-like, luminal B, normal-like, luminal A, and HER2+ subtypes, respectively. Notably, it is observed that in specific modules, such as module A in the basal-like subtype, module B in the luminal A subtype, and module A in the normal-like subtype, multiple circRNAs interacting with a single mRNA gene. This finding indicates that, despite lower expression levels of circRNAs compared to other RNA molecules, circRNAs play crucial roles in regulating specific mechanisms in different breast cancer subtypes^48^.

### Enrichment analysis

Enriched GO terms and pathways associated with the modules in $$ LC - MODUL^{C}$$, for each subtype of cancer $$ C$$ are analyzed to identify their involvement in specific pathways and processes. The significant processes enriched by these modules in each breast cancer subtype can be categorized into three major groups: cancer-related processes, immune-related processes, and oncogenic signaling pathways in cancer.

Table 2 displays the cancer-related processes and oncogenic signaling pathways in cancer derived from the modules in $$LC - MODUL^{C}$$ for each breast cancer subtype $$ C$$, while Table 3 illustrates immune-related processes derived from modules for each breast cancer subtype $$ C$$.

**Table 2:** Top pathways and GO enrichment terms of modules $LC - MODUL^{C}$ related to breast cancer subtype $C$.

Subtype ${\text{C}}$	Module’s name	Type of RNAs in module	Go terms or pathways	FDR
Basal-like	D (Fig. 5)	lncRNA, mRNA	Breast cancer	0.03041
Luminal A	E (Fig. 8)	lncRNA, mRNA	Progesterone-mediated oocyte maturation	0.0497
Basal-like	A (Fig. 5)	circRNA, mRNA	Mammary gland development pathway—Involution (Stage 4 of 4)	0.01572
Luminal B	C (Fig. 6)	circRNA, mRNA	GO:0060658, nipple morphogenesis	0.01182

**Table 3:** Top pathways and GO enrichment terms of modules $LC - MODUL^{C}$ related to the immune system in breast cancer subtype $C$.

Subtype $C$	Module’s Name	Type of RNAs in module	Go terms or Pathways	FDR
HER2+	C (Fig. 9)	lncRNA, mRNA	GO:2000409, positive regulation of T cell extravasation	0.03519
Luminal B	F (Fig. 6)	lncRNA, mRNA	B cell receptor signaling pathway	0.03837
Luminal B	G (Fig. 6)	lncRNA, mRNA	GO:0033083, regulation of immature T cell proliferation	0.04753
Luminal B	H (Fig. 6)	lncRNA, mRNA	GO:0042110, T cell activation	0.01656
Normal-like	B (Fig. 7)	lncRNA, mRNA	Immunoregulatory interactions between a Lymphoid and a non-Lymphoid cell	0.04297
Normal-like	B (Fig. 7)	lncRNA, mRNA	Adaptive Immune System	0.04297
Normal-like	B (Fig. 7)	lncRNA, mRNA	T Helper Cell Surface Molecules	0.04297
Basal-like	A (Fig. 5)	circRNA, mRNA	T Helper-2 activation	0.03637
Luminal A	A (Fig. 8)	circRNA, mRNA	GO:0002819, regulation of adaptive immune response	0.03149
Luminal A	C (Fig. 8)	circRNA, mRNA	GO:0002286, T cell activation involved in immune response	0.02239
Luminal A	D (Fig. 8)	circRNA, mRNA	T cell activation	0.0419
Luminal B	B (Fig. 6)	circRNA, mRNA	GO:0002765, immune response-inhibiting signal transduction	0.04507
Luminal B	C (Fig. 6)	circRNA, mRNA	GO:1905399, regulation of activated CD4-positive, alpha–beta T cell apoptotic process	0.009291
Luminal B	E (Fig. 6)	circRNA, mRNA	CD40/CD40L signaling	0.07089
Normal-like	A (Fig. 7)	circRNA, mRNA	B cell receptor signaling pathway	0.02161

**Figure 10:**
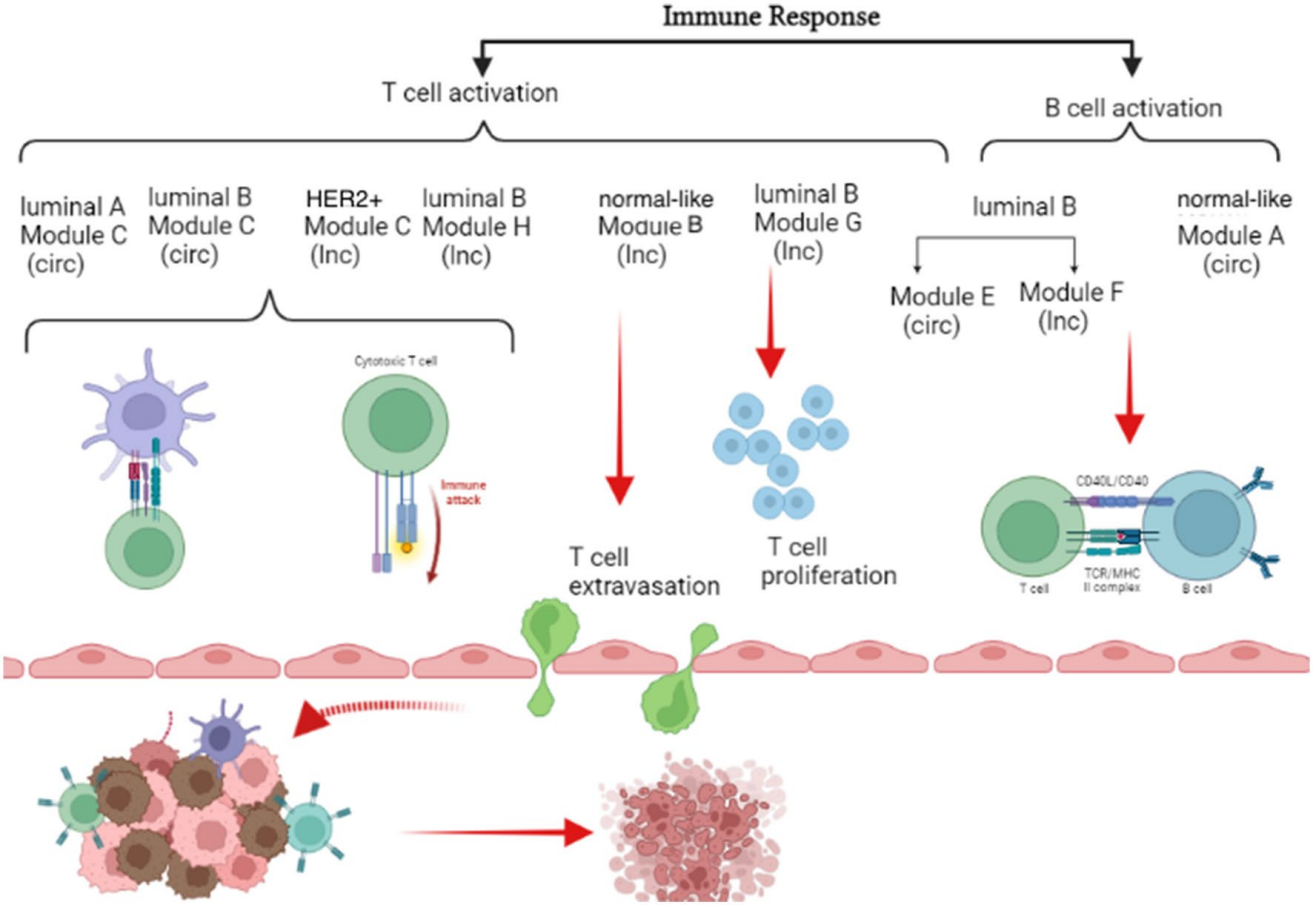
T cell and B cell activation processes in breast cancer subtypes. This figure represents the processes in which T cells and B cells are activated by the detected modules in breast cancer subtypes, with “lnc” and “circ” standing for lncRNA and circRNA, respectively. *Note*: The picture was created using biorender software (biorender.com).

Notably, the enrichment analysis indicates that the immune response primarily involves T cell and B cell activation processes separately. Figure 10 shows the corresponding modules for T cell and B cell activation in each of the five subtypes. It is noteworthy that all five subtypes possess a distinct module for activating T cells, while only luminal B and normal-like subtypes have specific modules related to B cell activation, emphasizing the importance of T cells over B cells in breast cancer subtypes.

### Biomarker detection

Through module enrichment, we establish the reliability of these modules for identifying biomarkers. In the following sections, we employ survival analysis and differential gene expression to discover biomarkers within these modules.

#### Survival analysis

**Figure 11:**
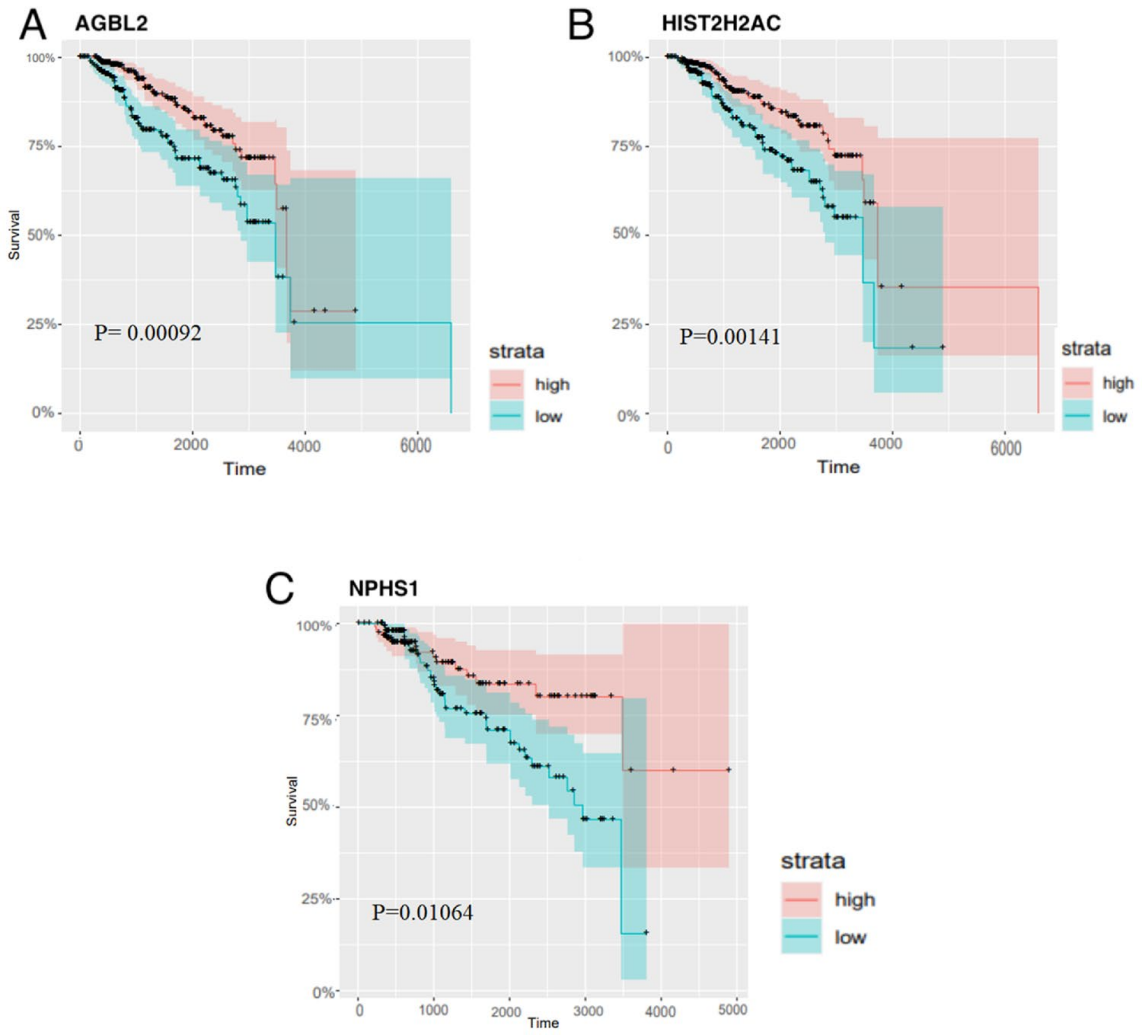
Kaplan–Meier survival curves for candidate mRNAs with lowest Log-rank test *p* values for (**A**, **B**) luminal B subtype and (**C**) normal-like subtype. Patients are divided into high-risk and low-risk groups based on the gene expression of these genes. The *p* value of the Log-rank test statistic has also been shown in each diagram*.*

We identify potential biomarkers from the mRNAs within the modules of $$ LC - MODUL^{C}$$ for each subtype $$C$$ using survival analysis. Among the identified genes, three mRNAs, AGBL2, HIST2HAC, and NPHS1, show a significant association with the overall survival of breast cancer patients, with Log-rank test *p *values of < 0.05. These results suggest that AGBL2 and HIST2HAC are significant prognostic biomarkers for luminal B subtype patients, while NPHS1 holds prognostic value for normal-like subtype patients. Independent KM survival curves for AGBL2, HIST2HAC, and NPHS1 genes are depicted in Fig. 11. In the subsequent sub-section, the analysis of differentially expressed genes further confirms the potential of NPHS1 as a biomarker. The Log-rank test *p *value for all genes in the $$LC - MODUL^{C}$$, for each subtype $$C \in BCS$$ can be found in Supplementary File S2.

#### Differential gene expression analysis

The common significantly differentially expressed genes between subtype $$C$$ and the other subtypes are documented in the set $$ Bio\_DEG^{C}$$ as the potential genes for biomarkers. The genes expressed differently across the five subtypes are presented in Supplementary Files S3, S4, S5, S6, and S7. Table 4 displays the gene names for each subtype $$C$$ in the $$Bio\_DEG^{C}$$ set, along with the average values of absolute logFC and FDR for differentially expressed genes between subtype $$ C$$ and the other subtypes. Additionally, standard derivations of logFC and FDR of differentially expressed genes between subtype $$C$$ and the other subtypes are provided in this table.

**Table 4:** The common significantly differentially expressed genes between subtype $C$ and the other subtypes are documented in the set $Bio\_DEG^{C}$ as the potential genes for biomarkers.

Subtype $C$	$Bio\_DEG^{C}$	avg_|logFC|	std_|logFC|	avg_FDR	std_FDR
Basal-like	circ_001845	0.909275	0.131957831	0.0234475	0.025229302
	circ_001124	0.855975	0.232192239	0.024875	0.025912079
	circ_003925	0.76815	0.001815673	0.0158925	0.023758302
	circ_000736	0.710225	0.107696406	0.028525	0.025866758
	circ_003996	0.762175	0.001776467	0.0158875	0.023748417
Luminal B	circ_00306	0.928925	0.214181813	0.0132075	0.025267296
	circ_00128	1.0188	0.126878236	0.0096	0.013684541
Normal-like	circ_000709	0.80905	0.012791794	0.009645	0.007470718
	NPHS1	0.717425	0.177538191	0.0218425	0.021153169
Luminal A	CAMKV	0.93575	0.467635994	0.0137	0.019086994
	circ_001855	0.5703	0.014347822	0.0448	0.02248733
HER2+	circ_00128	1.79915	0.523564581	0.003755	0.007230682
	circ_00173	1.85335	0.351477837	0.0001825	0.000279568

It is noteworthy that each of the five subtypes includes at least one circRNA gene considered as a biomarker. The negative values of logFC values shows all selected circRNA genes are down-regulated in the pairwise differential expression analysis between the five subtypes.

**Figure 12:**
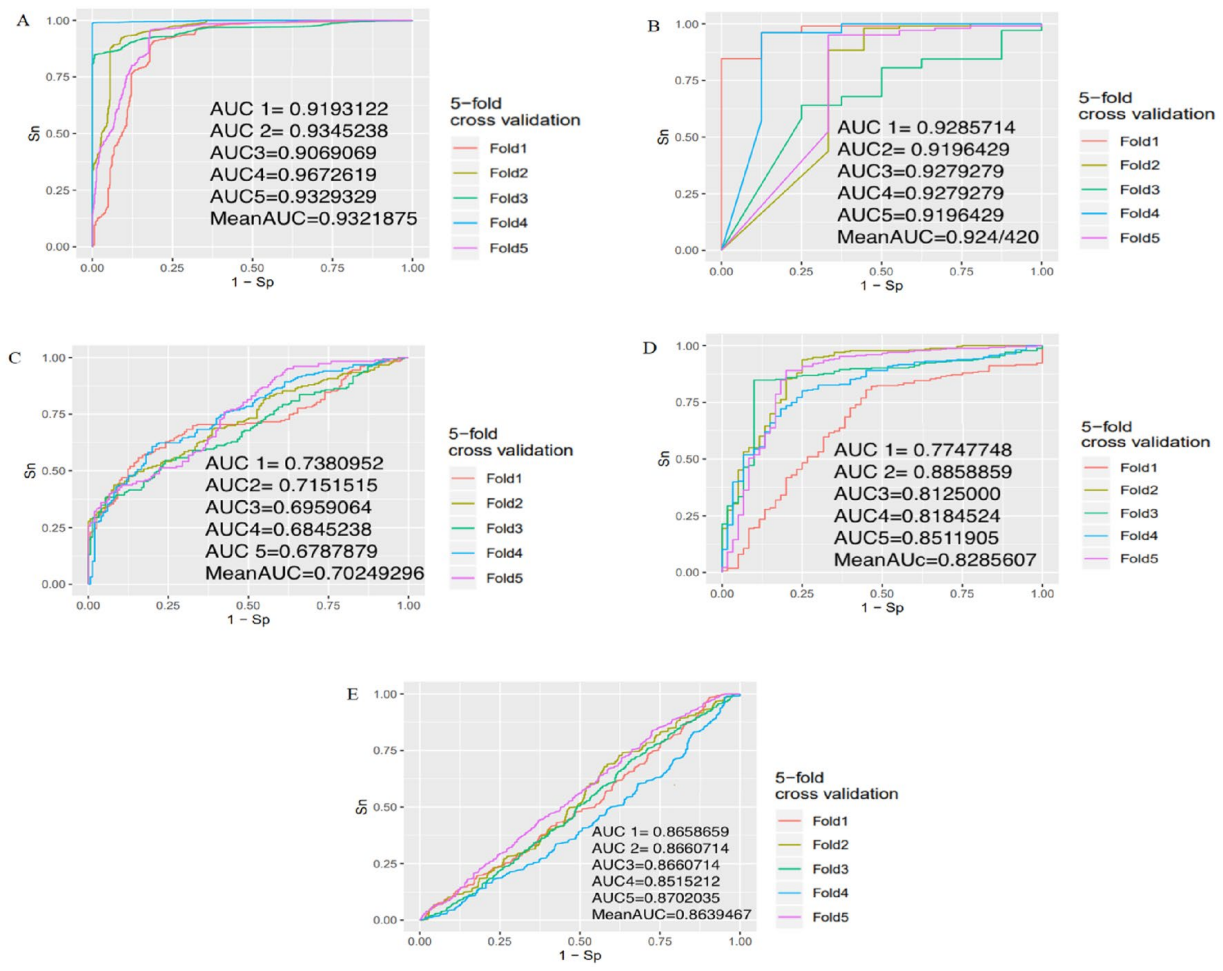
The validation results of selected genes in the set $Bio\_DEG^{C}$ for subtype $C$ using SVM which is (**A**) basal-like, (**B**) HER2+, (**C**) luminal A, (**D**) luminal B, and (**E**) normal-like. The AUC values for fivefold cross-validation are provided for each subtype, and the mean AUC values of fivefold cross-validation are also included*.*

In the following, to evaluate the selected genes in Table 4 as effective biomarkers for each breast cancer subtype, we design a predictor for each subtype using selected genes as described in the last step of the pipeline. Figure 12 presents the corresponding ROC curves and AUC values for each subtype.

The validation results of the model on each breast cancer subtype demonstrate satisfactory performance. The average AUC values of fivefold for basal-like, HER2+, luminal A, luminal B, and normal-like are obtained 0.93, 0.92, 0.70, 0.82, and 0.86, respectively. Overall, these results indicate that the selected genes in the set $Bio\_DEG^{C}$ for each subtype $C$ serve as robust biomarker sets for distinguishing between breast cancer subtypes.

Among the biomarkers predicted in this stage, circRNAs and lncRNAs emerge as promising candidates for breast cancer subtype prediction.

## Discussion

In the realm of immuno-oncology, a field at the intersection of immunology and cancer research, the development of potent immunotherapy treatments holds promise for various cancer types^49^. Understanding the immune system’s intricacies is paramount to advancing these treatments. Breast cancer research has underscored the pivotal role of T cells in tumor progression, across various subtypes^50–52^. This section investigates T cell regulation and activation mechanisms within these subtypes, revealing at least one module in each subtype enriched in these processes. as follows:

- In the HER2+ subtype, module C in Fig. 9 comprises ZDHHC14 and CROT mRNA genes, along with the lncRNA RGS5-AS1. This module is associated with the positive activation of T cells, which is influenced by ZDHHC14 and CROT mRNA genes. These genes are subject to regulation by the lncRNA RGS5-AS1 through hsa-miR-20a-5p. Additionally, it’s worth noting that ZDHHC14 is known as a tumor suppressor gene with relevance across various cancer types^53^.
- In the luminal B subtype, modules G and H in Fig. 6 are pivotal in regulating T cell proliferation and activation, while module C is associated with nipple morphogenesis and influences the T cell apoptotic process. These modules reveal the presence of hsa-miR-149-3p and hsa-miR-133a-3p miRNAs, which are shared between lncRNA C6orf223 and the mRNA molecules in module G, and between lncRNA HHLA3-AS1 and the mRNA molecules in module H. This discovery underscores the potential of these miRNAs as influential genes involved in suppressing various oncogenic pathways^54^. As a result of our analysis, we suggest that biologists consider lncRNAs C6orf223 (in module G) and HHLA3-AS1 (in module H), as well as circ_000349 (in module C), as potential biomarkers for the luminal B subtype.
- In the basal-like subtype, module A (see Fig. 5) includes BCL2L12 mRNA linked to T Helper-2 (Th2) activation processes. This gene’s role in modulating immune responses suggests it as a potential therapeutic target^55^. CircRNAs, circ_003996, circ_003925, circ_004109, and circ_000736, in this module further regulate BCL2L12 via hsa-miR-539-5p.
- In the luminal A subtype, circ_002665 (module C in Fig. 8) and circ_001855 (module D in Fig. 8) appear to play essential roles in regulating T-cell activation mechanisms. These circRNAs are involved in the regulation of HOXB6 in Module C and HLA-F and CAMKV in Module D. Previous studies have discussed the roles of HOXB6 in breast cancer^56^, and the dysregulation of HLA-F and CAMKV has been reported in various cancers^57–59^. This analysis underscores the precise involvement of HOXB6, HLA-F, and CAMKV in tumor progression through T cell activation processes in the luminal A subtype.
- In the normal-like subtype, module B (Fig. 7), which is enriched in T Helper Cell Surface Molecules, includes DLEU1. DLEU1 has been identified as an up-regulated lncRNA in breast cancer tissues and cells, particularly in tumors with high malignancy^60,61^. This lncRNA interacts with NPHS1 mRNA. Based on our analysis, NPHS1 exhibits significant prognostic value and shows differential expression between normal-like subtype and the other four breast cancer subtypes. This underscores NPHS1 as a potential dependable immune molecular marker for normal-like subtype. Interestingly, three shared miRNAs, namely hsa-miR-378a-5p, hsa-miR-125a-3p, and hsa-miR-150-5p, are found to exist between DLEU1 and NPHS1. These miRNAs are known as anti-apoptotic agents in breast cancer, further highlighting their potential roles in immune regulation within the normal-like subtype^62–64^.

These findings emphasize the complex interplay between various RNA molecules and immune mechanisms across breast cancer subtypes, offering insights into potential therapeutic targets and diagnostic markers.

## Conclusion

In conclusion, breast cancer poses a significant global health challenge. Recent research highlights the critical roles of ncRNAs, specifically lncRNAs and circRNAs, in both cancer and immune system functions. The competing endogenous RNA hypothesis provides a valuable framework to understand the interplay between mRNAs and ncRNAs in the context of miRNA regulation.

This study employed an innovative pipeline named Pre_CLM_BCS to investigate the roles of ncRNAs in various breast cancer subtypes. By constructing subtype-specific ceRNA networks, we unveiled distinct modules associated with immune and cancer-related processes within each subtype. Table 5 reveals the results of our pipeline, which has allowed us to identify several potential biomarkers specific to different breast cancer subtypes. Additionally, our analysis has unveiled certain ncRNAs that appear to play pivotal roles in the progression of their respective breast cancer subtypes, possibly through their regulatory effects on T cell mechanisms.

**Table 5:** A compilation of potential biomarkers identified by our pipeline.

Subtype	Biomarkers	
	Cancer-related processes	Immune-related processes
Basal-like	circ_001845	circ_003996
	circ_001124	circ_003925
	circ_003925	
	circ_000736	
	circ_003996	
Luminal B	circ_00306	C6orf223
	circ_00128	HHLA3-AS1
		circ_000349
Normal-like	circ_000709	DLEU1
	NPHS1	
Luminal A	CAMKV	circ_002665
	circ_001855	circ_001855
HER2+	circ_00128	RGS5-AS1
	circ_00173	

In the realm of immuno-oncology, understanding T cell regulation and activation mechanisms is essential. This study revealed the involvement of specific RNA molecules in these processes across different breast cancer subtypes. These findings provide insights into potential therapeutic targets and diagnostic markers, shedding light on the intricate interplay between various RNA molecules and immune mechanisms in the context of breast cancer subtypes. This research contributes to the ongoing efforts to improve breast cancer diagnosis and treatment, ultimately reducing the global burden of this disease.

While we have developed a pipeline to investigate the competitive regulatory mechanisms of circRNAs and lncRNAs within the context of miRNAs competition for the detection of breast cancer subtypes, it’s evident that each step of the pipeline has potential for improvement.

In our future research, we aim to explore the implementation of graph neural networks (GNNs)^65^ to enhance the construction of specific ceRNA networks in the third step of the pipeline. GNNs have shown promise in improving the accuracy of network analysis and prediction, and we anticipate that leveraging this technology will lead to the discovery of more effective biomarkers.

Furthermore, we plan to investigate the integration of ceRNA networks with gene-protein signaling networks^66–68^. This combination promises to provide a more holistic view of the regulatory interactions within a biological system. By unifying coding and ncRNA interactions with protein signaling pathways, we can gain a deeper understanding of how these components mutually influence each other’s functions, ultimately shedding light on complex biological processes and diseases. This integrated approach holds the potential to identify key regulatory nodes, uncover novel biomarkers, and elucidate the intricate interplay between ceRNA networks and protein signaling.

### Supplementary Information

Supplementary Information 1.

Supplementary Information 2.

Supplementary Information 3.

Supplementary Information 4.

Supplementary Information 5.

Supplementary Information 6.

Supplementary Information 7.

## Data Availability

The study utilized miRNA-seq and RNA-SeqV2 level 3 data, which encompass mRNA and lncRNA expression data of breast cancer, sourced from TCGA Research Network (http://cancergenome.nih.gov/) using the National Cancer Institute (NCI) Genomic Data Commons (GDC) resource (https://gdc.cancer.gov/). CircRNA expression data for breast cancer was acquired based on the information provided by Asha et al. (https://doi.org/10.18632/oncotarget.13134)^28^.
