# Supplementary figures and images for: Exploring ceRNA networks for key biomarkers in breast cancer subtypes and immune regulation
Source: Sci Rep. 2023 Nov 27;13:20795. doi: 10.1038/s41598-023-47816-z (PMC10682442; doi:10.1038/s41598-023-47816-z)

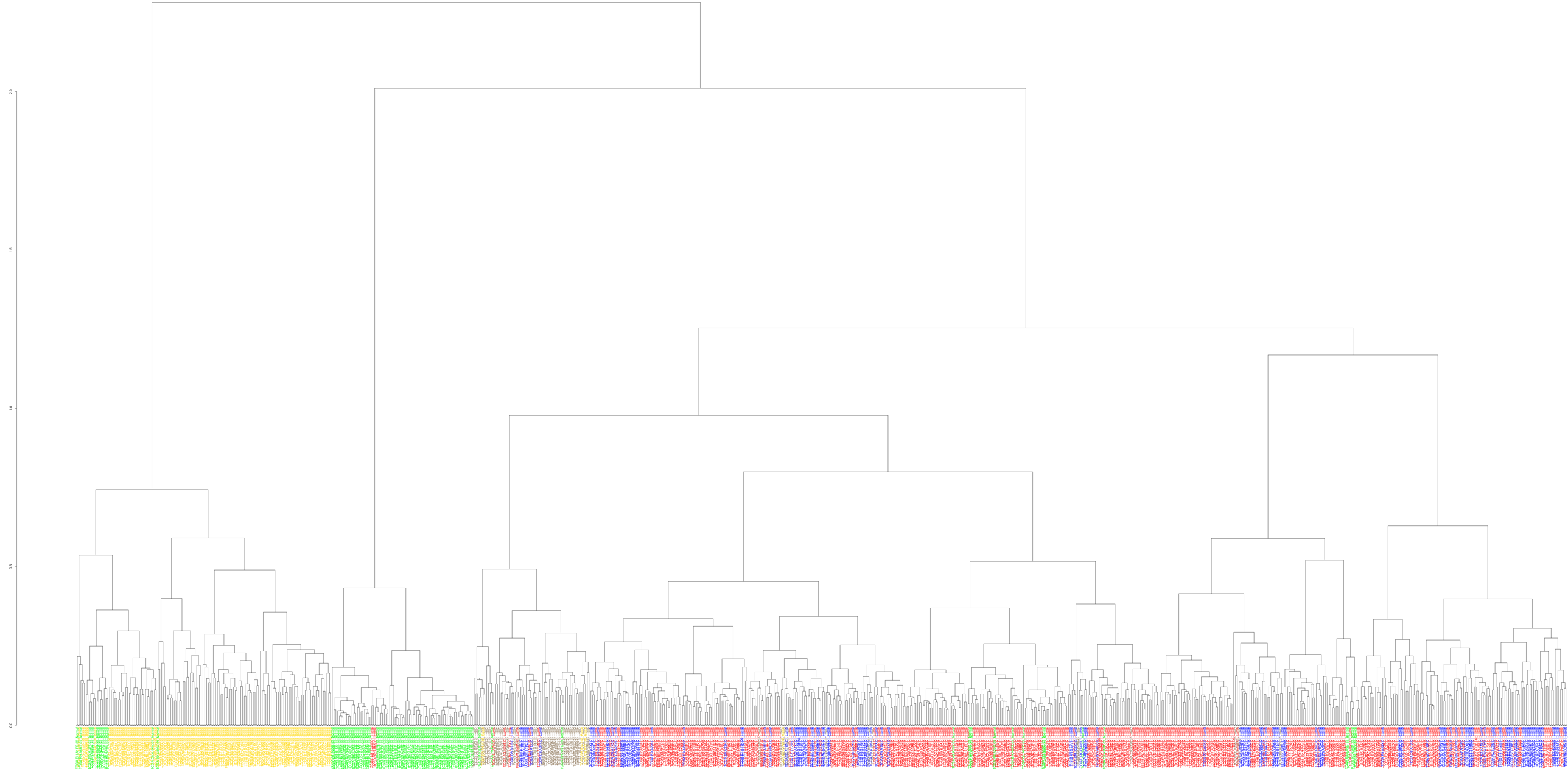

Supplement: Supplementary file 1 — Supplementary Information 1. [file 41598_2023_47816_MOESM1_ESM.pdf]
